# The use of radiomic analysis of magnetic resonance imaging findings in predicting features of early osteoarthritis of the knee—a systematic review and meta-analysis

**DOI:** 10.1007/s11845-024-03714-5

**Published:** 2024-06-01

**Authors:** Martin S. Davey, Matthew G. Davey, Paddy Kenny, Adrian J. Cassar Gheiti

**Affiliations:** 1 Connolly Hospital Blanchardstown, Dublin, Ireland; 2 National Orthopaedic Hospital Cappagh, Dublin, Ireland; 3 Royal College of Surgeons in Ireland, Dublin, Ireland

**Keywords:** Cartilage, Knee, MRI, Osteoarthritis, Radiomics

## Abstract

The primary aim of this study was to systematically review current literature evaluating the use of radiomics in establishing the role of magnetic resonance imaging (MRI) findings in native knees in predicting features of osteoarthritis (OA). A systematic review was performed with respect to PRISMA guidelines in search of studies reporting radiomic analysis of magnetic resonance imaging (MRI) to analyse patients with native knee OA. Sensitivity and specificity of radiomic analyses were included for meta-analysis. Following our initial literature search of 1271 studies, only 5 studies met our inclusion criteria. This included 1730 patients (71.5% females) with a mean age of 55.4 ± 15.6 years (range 24–66). The mean RQS of included studies was 16.6 (11–21). Meta-analysis demonstrated the pooled sensitivity and specificity for MRI in predicting features of OA in patients with native knees were 0.74 (95% CI 0.71, 0.78) and 0.85 (95% CI 0.83, 0.87), respectively. The results of this systematic review suggest that the high sensitivities and specificity of MRI-based radiomics may represent potential biomarker in the early identification and classification of native knee OA. Such analysis may inform surgeons to facilitate earlier non-operative management of knee OA in the select pre-symptomatic patients, prior to clinical or radiological evidence of degenerative change.

## Introduction

Osteoarthritis (OA) represents the most commonly found age-related degenerative joint disorder, which affects over 10% of the world’s population, and has previously been shown to cause significant joint pain, stiffness and mobility decline [1, 2]. The pathophysiology of OA has been extensively studied and has been demonstrated to affect all tissues in the joint, resulting in the degeneration of articular cartilage in progressive fashion followed by the degeneration of subchondral bone [3]. Although x-rays remain the mainstay of imaging in patients with OA [4], many patients show early signs of OA on magnetic resonance imaging (MRI) at an earlier stage, often before symptoms ensue, particularly at a young age [5].

Radiomics analysis is a promising, emerging radiological tool, which allows evaluation of primary diagnostic medical imaging to build phenotypic signatures of the pathogenesis of particular conditions or diseases that are imperceptible to the human eye, therefore potentially allowing detailed visual characterization of a prospectively developing disease [6]. Therefore, the utility of radiomic analysis has the potential to enhance existing information available to clinicians from current imaging, through non-intuitive mathematical assessment of underlying disease properties, many of which are undetectable to even the most experienced fellowship-trained radiologists [7].

Diagnostic modalities to predict OA represent a significant unmet need in patients with ongoing knee pain despite ‘normal’ knee radiographs and may facilitate personalized management algorithms for patients presenting with potential early native knee OA [8]. Theoretically, radiomic techniques may be able to predict potential disease presence prior to extensive clinical progression of symptoms in patients who have undergone higher order imaging [6, 9]. The primary aim of this study was to systematically review current literature evaluating the use of radiomics in establishing the role of MRI findings in native knees in predicting features of OA. The secondary aim of this study was to characterize the sensitivity and specificity of MRI in diagnosing potential OA in native knees.

## Methods

### Pre-study planning

Prior to study commencement, the investigators registered this study with PROSPERO (registration number *CRD42023413688*) with a view to answering the research question as described in the "PICO" section below. Following the Preferred Reporting Items for Systematic Reviews and Meta-Analyses (PRISMA) guidelines [10], the investigators performed this systematic review in accordance with the AMSTAR 2 criteria [11] alongside the Cochrane Handbook for Systematic Reviews of Diagnostic Test Accuracy [12].

### PICO

Population: patients who have undergone MRI of their native knee with suspicion of potential OA (in the absence of findings of OA on plain film radiographs).

Intervention: the discriminative ability of radiomic cartilage analyses (i.e. artificial intelligence in the form of machine learning techniques) of MRI of their affected native knee to predict subsequent OA.

Comparison: the ability of radiomic analyses in evaluating native knee cartilage MRI in predicting subsequent knee OA.

Outcomes: the assessment of the utility of radiomic imaging using MRI in the prediction of native knee OA, with sensitivity and specificity analyses.

### Definitions

- ‘Osteoarthritis’ was defined with respect to the previously established Delphi exercise definition of tibiofemoral OA [13]; *The presence of both group [A] features or one group [A] feature and two or more group [B] features: Group [A] after exclusion of joint trauma within the last 6 months (by history) and exclusion of inflammatory arthritis (by radiographs, history and laboratory parameters): i) Definite osteophyte formation, ii) Full thickness cartilage loss. Group [B]: i) Subchondral bone marrow lesion or cyst not associated with meniscal or ligamentous attachments ii) Meniscal subluxation, maceration or degenerative (horizontal) tear iii) Partial thickness cartilage loss (where full thickness loss is not present) iv) Bone attrition.*
- ‘Radiomic analysis’ was defined as representing artificial intelligence in the form of any of the following: (1) machine learning techniques, (2) conventional neural networks or (3) deep brain learning in analyzing findings on MRI.

### Search strategy

Using PRISMA guidelines, two independent reviewers (M.S.D. and M.G.D.) carried out a search for relevant studies of the PubMed, Embase and Scopus databases in April 2023. Prior to investigation commencement, it was pre-determined that no time limit would be applied to the search. The following search terms were applied to each of the aforementioned electronic databases: (osteoarthritis or arthritis or degeneration) and (radiomics). After manually removing all duplicate studies, both investigators examined the titles and abstracts of all potential studies identified from the literature search whilst applying our exclusion criteria. It was pre-determined that in cases of discrepancies in opinion between the reviewers with respect to an excludable study, a third author (A.C.G.) would act as an arbitrator; however, this was not required. Thereafter, both reviewers independently evaluated the full texts of all potentially eligible studies using pre-determined inclusion criteria. Finally, both reviewers performed a final screening of reference lists of all studies that met the inclusion criteria.

### Inclusion and exclusion criteria

Prior to the commencement of the search, all investigators agreed on the pre-determined inclusion and exclusion criteria specific to the index study. The inclusion criteria included the following: (1) studies with patients with confirmation of a diagnosis of knee OA on MRI; (2) studies where patients had undergone radiomic tissue analysis of diagnostic MRI of their native knee prior to surgical intervention for OA; (3) studies reporting the sensitivity, specificity or receiver operating characteristic (ROC) curve analysis in the evaluation of the radiomic testing reliability in the prediction of OA features on MRI; (4) studies printed in the English language; (5) acceptance in a peer-reviewed journal. The exclusion criteria included the following: (1) studies reporting outcomes of patients with OA at a site other than knee OA; (2) studies which performed radiomic analysis of computed tomography (CT), ultrasound (US) or x-ray (XR) imaging; (3) non-clinical studies; (4) case reports or series with less than 5 patients.

### Data extraction and quality assessment

As outlined prior, two independent reviewers carried out this literature search using the search strategy described in the "Search strategy" section. A review of all potential studies was performed independently by both reviewers to allow consensus for each study to as whether all inclusion criteria were met, before the extraction of data on a pre-determined data collection sheet previously designed by the senior author. Outcomes of interest for data extraction included the following: (1) study design, (2) country of origin, (3) level of evidence (LOE), (4) number of patients, (5) knee side and (6) sensitivity, specificity or ROC analyses. The criteria outlined by Lambin et al. [14] were used for evaluation of the quality of included radiomic studies using the ‘Radiomics Quality Score’ (RQS).

### Statistical analysis

Descriptive statistics presented clinical and radiological data as proportions for analysis. For each study, specific estimates of sensitivity and specificity were calculated from published study reportings. Using Review Manager (RevMan), version 5.4 (*Nordic Cochrane Centre, Denmark*), summary ROC analysis was used to illustrate the relationship between sensitivity and specificity of imaging modality radiomic analysis with respect to knee OA confirmed on MRI and to convey the diagnostic test performance of each imaging modality. Additionally, the authors chose to report 95% confidence intervals (CI) for all sensitivity and specificity analyses, with a *P* < 0.05 being considered statistically significant.

## Results

### Literature search

**Fig. 1:**
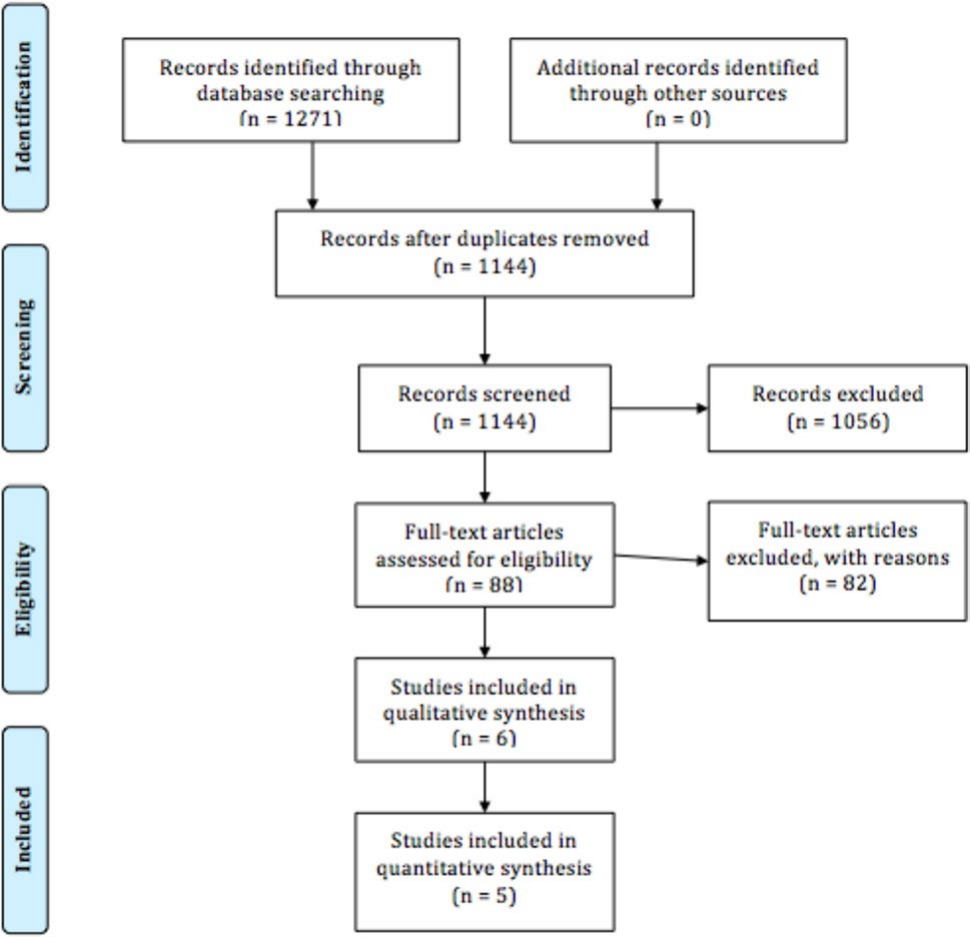
PRISMA flow chart

The overall literature search yielded a total of 1271 studies. Following the removal of 244 duplicates, the remaining 1027 studies were subject to our exclusion criteria. Subsequently, the application of our inclusion criteria to the remaining resulted in 5 studies (1730 patients) being included in total [15–19]. An additional study of 1174 patients was noted to perform radiomic analysis in predicting potential knee OA using plain radiographs, however was excluded on the basis of our aforementioned inclusion criteria. The PRISMA flow chart is illustrated in Fig. 1.

### Study characteristics, patient demographics and site of analysis

The 5 included studies included 1730 patients with a mean age of 55.4 ± 15.6 years (range 24–66), of which 1236 (71.5%) of whom were female. Of the included studies, 4 were performed in China, and 1 was carried out in The Netherlands. All included studies represented level III evidence, with a mean RQS of 16.6 (11–21). Additionally, 1 study analysed radiomic features of the infra-patellar fat pad (IPFP) on MRI, whilst 4 studies evaluated the tibiofemoral (TF) joint. Of these, 1 study focused on patients who had undergone anterior cruciate ligament reconstruction (ACLR). A total of 4 of the included studies were performed in China, and 1 was carried out in The Netherlands. The study characteristics and patient demographics of each study are described in Table 1.

**Table 1:** Patient demographics and study characteristics

Authors and publication yr	Country	*N* total	*N* females	Age (yrs)	F/U	BMI (kg/m^2^)	*N* OA	MRI (T)	OA area
Hirvasniemi (2021)	Netherlands	665	665	54.6	NR	26.8	76	1.5	TF
Yu (2023)	China	604	410	61.5	48	28.3	302	3	IPFP
Lin (2023)	China	216	101	64	24	29	216	1.5	TF
Xue (2022)	China	88	49	52.2	NR	24.6	56	3	TF
Xie (2021)	China	157	11	25	NR	23	157	3	TF

*BMI* body mass index, *FU* follow-up, *IPFP* infra-patellar fat pad, *Mo* months, *MRI* magnetic resonance imaging, *N* number, *OA* osteoarthritis, *T* tesla, *TF* tibiofemoral, *Yrs* years

### MRI radiomics

**Fig. 2:**
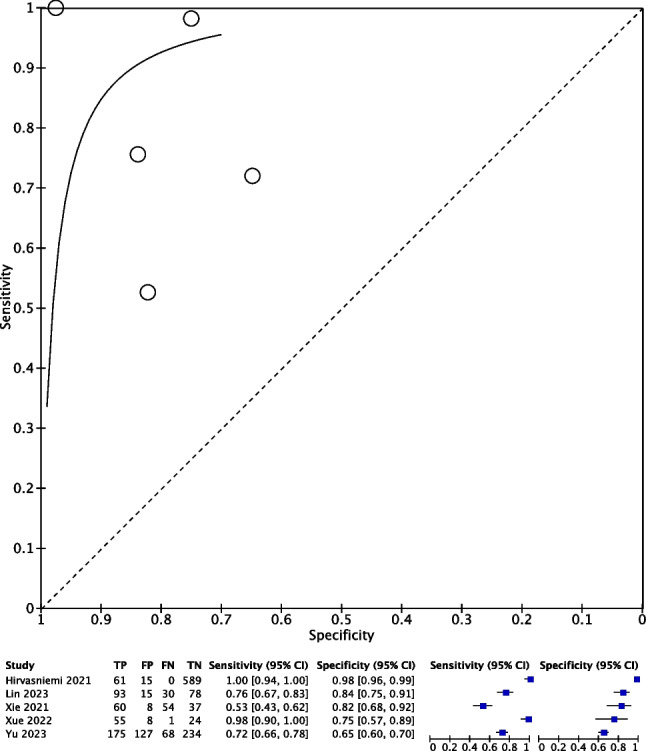
Meta-analysis of radiomic analysis in predicting knee OA from MRI with sensitivity and specificity analysis

**Fig. 3:**
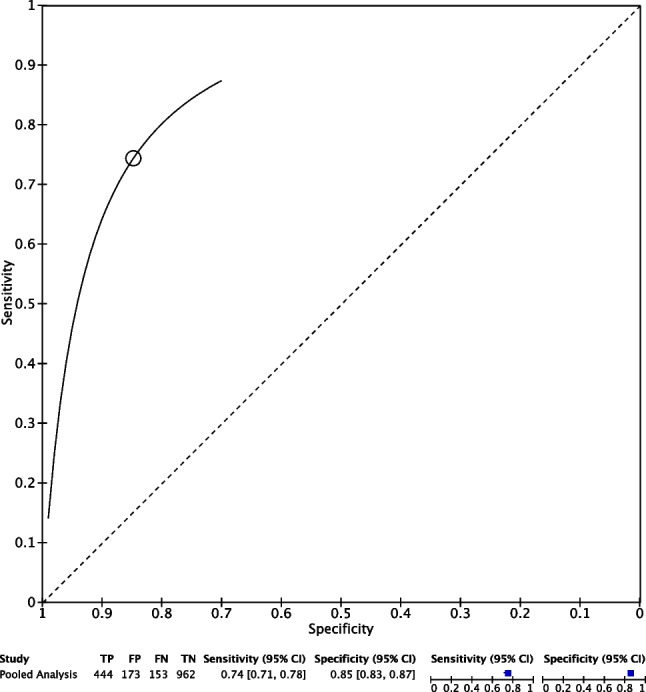
Meta-analysis of radiomic analysis in predicting knee OA from MRI with pooled sensitivity and specificity analysis

All 5 studies reported radiomic analysis for predicting OA in native knees of 1730 patients’ MRIs using radiomic analysis. None of the included studies utilized MRI with contrast. The overall pooled sensitivity and specificity for MRI in predicting features of OA in patients with native knees were 0.74 (95% CI 0.71, 0.78) and 0.85 (95% CI 0.83, 0.87), respectively. A summary of radiomic data for each study is described in Table 1. Further data in the form of forest plots depicting the meta-analysis of radiomic analysis in predicting OA from knee MRIs and pooled sensitivities and specificities are seen in Figs. 2 and 3.

## Discussion

The most important finding in this meta-analysis is that emerging radiomic analysis results in high sensitivities and specificities for MRI in the potential prediction of cases of early knee OA in the pre-symptomatic patient. As the quality and quantity of current evidence with respect to radiomic analysis of MRI studies in predicting potential knee OA studies are limited at present, to suggest its routine adoption into clinical practice would be dubious. However, the data presented in this study proposes that the use of radiomic analysis may have discriminative potential to identify select patients whom sub-clinical early OA may be managed in a proactive manner. Should prospective datasets be generated and subsequently analysed by international biotechnological corporations, the true role of radiomic analysis of MRI in these patients may represent a potential opportunity for more personalized treatment strategies, including tailored preventative, proactive management of the asymptomatic patient likely to develop knee OA in the not too distant future.

Knee OA is an increasingly common cause of significant morbidity in the twenty-first century, with orthopaedic surgeons performing an ever-increasing number of total knee arthroplasties (TKAs) on an annual basis [20]. As OA represents not only a disease of cartilage but bone and surrounding soft tissues [3], MRI represents a potential imaging modality which may show surgeons an accurate depiction of both the soft and bony structures of the affected joint; this may subsequently allow potential detection of patients with early progression of knee OA [4]. In their study of 244 knees, MacKay et al. previously reported that MRI analysis of tibial subchondral bone allows early radiological detection of knee OA at chronological intervals as short as 36 months [8].

Radiomic analysis and machine learning techniques have emerged as an increasingly accessible radiological tool being utilized across a variety of medical specialties in hope of predicting and characterizing potential patient outcomes and prognoses [21]. Subsequently, radiomic analysis has been utilized in oncological practice in order to evaluate primary tumour imaging in hope of aiding prediction of potential metastatic deposits [22–24]. This has been furthermore applied to orthopaedic oncology, with Pereira et al. reporting their outcomes of predicting the presence of distant disease in patients with osteosarcoma identified on computed tomography (CT) [25], and Gitto et al. using machine learning to establish diagnosis of atypical cartilaginous tumours from chondrosarcomas [26]. However, little radiomic analysis in the patient at risk of developing osteoarthritis is available to date.

A total of 4 of the 5 included studies reported outcomes of radiomic analysis of the TF joint, particularly focusing on radiological features predictive of medial joint OA [15–18]. In their study of 88 patients post-ACLR, Xie et al. reported a specificity of 0.98 in radiomic analysis of knee MRIs in the subsequent 4 years [17]. However, the authors acknowledge that this study contains the smallest cohort of included patients, the majority of which had prior ACL reconstruction prior to undergoing MRI. These results are much more specific than those of the 1730 patient population of this review, with meta-analysis demonstrating a pooled specificity of 0.85 overall. Yu et al. reported their outcomes of machine learning analysis of the IPFP in patients with native knees, demonstrating specificities and sensitivities of 0.56 and 0.80, respectively, in the development of subsequent OA at 12-month follow-up [19]. This suggests that perhaps potential radiomic analysis of soft tissue on MRI may possess a role in ruling out potential knee OA in the following year, with the caveat of 1 in 5 receiving a false-negative result.

### Limitations

This investigation found high sensitivities and specificities in potential prediction capacity of radiomic analysis of MRI for OA of the native knee; however, the authors acknowledge that further prospective larger studies evaluating outcomes are warranted. Moreover, the heterogeneity in reporting amongst included studies is recognized as representing an apparent limitation, including the different MRI scanners utilized between the various included studies. Although radiomics may offer a novel prediction method for surgeons in the identification and classification of knee OA in select patients, it should be noted that all 5 included studies represent low levels of evidence and include both cohorts of training and validation sets. Therefore, the orthopaedic literature should potentially welcome any proposal of prospective studies with the aim to further establish and quantify the role of radiomics in the detection of OA in the asymptomatic patients following MRI, to further build on the potentially promising results established in this study.

## Conclusion

The results of this systematic review suggest that the high sensitivities and specificity of MRI-based radiomics may represent potential biomarker in the early identification and classification of native knee OA. Such analysis may inform surgeons to facilitate earlier non-operative management of knee OA in the select pre-symptomatic patients, prior to clinical or radiological evidence of degenerative change.
